# Effects of an online home-based exercise intervention on sleep quality in breast cancer patients during the COVID-19 lockdown: a pilot study

**DOI:** 10.1007/s00520-025-09710-4

**Published:** 2025-07-01

**Authors:** Lucía Sagarra-Romero, Raquel Val-Ferrer, Ángel I. Fernández-García, Enrique Ramón-Arbués

**Affiliations:** 1 Faculty of Health Sciences, Universidad San Jorge, Autovía A-23, Km 299, Villanueva de Gállego, 50830 Zaragoza Spain; 2 GAIAS Research Group, Facultad de Ciencias de la Salud, Universidad San Jorge, Villanueva de Gállego, Zaragoza Spain; 3 Department of Physiatry and Nursing, Faculty of Health and Sport Science (FCSD), Ronda Misericordia 5, 22001 Huesca, Spain; 4 EXER-GENUD (Growth, Exercise, Nutrition and Development) Research Group, Universidad de Zaragoza, Zaragoza, Spain; 5 B53_23R: SAPIENF Research Group, Universidad San Jorge, Zaragoza, España

**Keywords:** Online exercise program, Breast cancer survivors, Lockdown, Sleep quality, Pittsburgh sleep quality index, Actigraphy device

## Abstract

**Purpose:**

During the COVID-19 pandemic, patients with cancer have been particularly vulnerable to experiencing high levels of psychological distress. This study implemented a 16-week online home-based exercise program to assess its impact on sleep quality during the pandemic. Fifteen participants completed the program, which included functional exercises conducted via Zoom. Sleep quality was evaluated using the Pittsburgh Sleep Quality Index (PSQI) and accelerometry.

**Results:**

Participants showed notable improvements: PSQI scores decreased significantly, indicating better sleep quality, reduced sleep latency, and increased sleep duration and efficiency. Objective measures also reflected improvements in total sleep time and reduced average awakening duration.

**Conclusions:**

Online exercise interventions can effectively enhance sleep quality in breast cancer survivors, emphasizing the importance of integrating exercise into their care, especially during disruptive periods like a pandemic.

## Introduction

The impact of COVID-19 on sleep patterns has received considerable attention. In fact, sleep disturbances have emerged as another consequence of the COVID-19 pandemic. The prevalence of sleep disturbances (including poor sleep quality and insomnia) is estimated to affect approximately 40% of the general population during this period [1].

In cancer survivors, poor sleep quality is indeed a common and distressing long-term effect. It is widely known that cancer and its treatment can have a significant effect on sleep quality and duration, leading to various sleep disturbances [2].

In the context of COVID-19, the impact of lockdown both in patients with active cancer and in cancer survivors has had a notable effect on their physical, psychological, and social well-being, increasing anxiety, catastrophic thoughts, and sleep disorders [3].

In the case of breast cancer, survivors tend to be more vulnerable to poor sleep quality than the general population is. In fact, sleep problems are among the most common long-term health issues faced by women with breast cancer [4].

Additionally, patients can experience sleeping problems (i.e., insomnia) even 10 years after diagnosis. This long-term consequence might have negative effects on quality of life, including physical distress, depression, or anxiety [5].

The COVID-19 pandemic and related mobility restrictions significantly impacted various aspects of daily life, including the ability to maintain regular physical activity. For women who are cancer survivors, this lack of exercise could exacerbate issues related to sleep quality and sleep disorders.

Emerging research has shown the positive effect that exercise can have on the quality of sleep in breast cancer survivors. For example, the National Institutes of Health (NIH) proposed that physical exercise (PE) may be an adjuvant intervention to improve sleep quality in cancer survivors [6]. Other studies have suggested an association between PE and a reduction in severe insomnia in breast cancer survivors [7, 8].

The purpose of this study was to assess the effects of an online home-based exercise intervention on sleep quality, sleep efficiency, and sleep patterns in breast cancer survivors during lockdown.

## Materials and methods

### Participants and recruitment strategy

This study is a quasi-experimental study with a pre-post-test design (no control group). Twenty-two female breast cancer survivors were selected for the study, 15 of whom completed our online exercise program (rejection/noncompliance rate 31.8%). All the participants belonged to the Association of Aragonese Women of Genital and Breast Cancer (AMACGEMA) of Zaragoza (Spain).

### Online home‑based exercise program

**Fig. 1:**
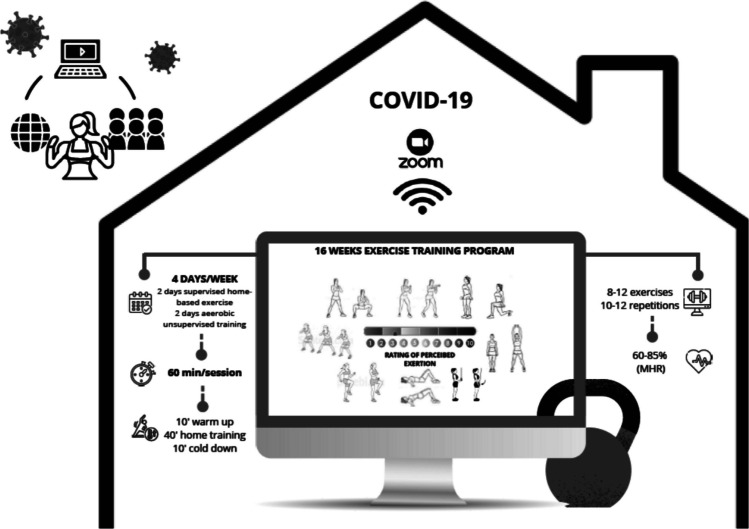
Online home-based exercise program characteristics

The program lasted 16 weeks (February–June 2020). The intervention included four weekly training sessions (two supervised functional training sessions and two unsupervised aerobic training sessions) (see Fig. 1).

In the supervised part, the trainer led the session by connecting online synchronously with the participants through the ZOOM application (Zoom Video Communications, Inc., 2020). During the connection, the participants were able to watch the performance, interact, provide comments, or ask questions. The trainer was also able to evaluate the participants’ performance and provide individual or group comments and corrections when necessary. The duration of the sessions was 60 min (10 min of warm-up, 40 min of the main part of the training, and 10 min of cool-down). Each session included a combined circuit of 8 to 12 functional exercises to improve strength and cardiorespiratory fitness (squats, split squats, walking lunges, calf raises, glute bridge, band dips, core, walking/jogging on the place, lateral step-up with the bicep curl or shoulder press, punches, and jumping jacks). The exercises involved body weight, elastic bands, or other materials available at home (e.g., water bottles). For the functional strength exercises, a protocol of 10 to 12 repetitions was developed, while the aerobic exercises lasted approximately 30 s. Two series were performed until week 12, and three series were performed from weeks 13 to 16. A minimum rest of 90 s was developed between exercises.

The participants were asked to wear a heart rate monitor during all the sessions. The work intensities ranged between 60 and 85% of the maximum heart rate (HRmax) obtained from the Rockport test and were calculated via the Karvonen formula [9].

Finally, to improve the specificity of the program and follow the WHO physical activity recommendations, we recommend that participants add two unsupervised home aerobic sessions every week (20–30 min/session; 60–85% HRmax). These aerobic sessions could include self-selected exercises, such as dance choreography, indoor cycling, and elliptical cycling.

### Measures

#### Subjective assessment of sleep

The Pittsburgh Sleep Quality Index (PSQI) was used to measure quality and patterns of sleep. The questionnaire consists of 24 questions that include items such as sleep duration; sleep-associated events such as difficulties starting to sleep; awakenings; nightmares; snoring; respiratory disturbances; quality of sleep; intake of sleeping medications; and the presence of daytime sleepiness. The sum of these components provides a global score (0–21) and indicates the quality of the general sleep of the person evaluated. The higher the total score is, the worse the quality of sleep. The PSQI is a validated tool for patients with breast cancer [10].

#### Objective assessment of sleep

Sleep quality and patterns were measured by accelerometry (ActiGraph GT9X Link; Actigraph, 49 E. Chase St. Pensacola, FL 32502), which is a widely validated and objective tool used to evaluate sleep quality in patients with breast cancer [11].

The participants wore accelerometers on their nondominant arms, secured with an elastic belt. The participants were instructed to keep the accelerometer on all day, including during sleep. The data were recorded at 60-s intervals and analyzed via ActiLife 6.0 software (ActiGraph, Pensacola, FL, USA).

### Statistical analysis

The descriptive data are presented as the means, standard deviations, and 95% confidence intervals for the pre- and post-intervention differences. Changes in the PSQI and actigraphy results were assessed via paired *t* tests or Wilcoxon tests depending on whether the data were normally distributed or not. Additionally, effect sizes were calculated using Hedges' g. All the statistical analyses were performed via the Statistical Package for the Social Sciences software (version 28.0; SPSS Institute Inc., Chicago, IL, USA). The accepted level of statistical significance was *p* < 0.05.

### Ethical considerations

The participants were informed in detail about the study protocol, objectives, and procedures. Those who voluntarily agreed to participate signed a consent report. The study was carried out on the basis of the ethical principles of the Declaration of Helsinki and the Good Clinical Practice Guide. The project was approved by the Ethics Committee of the Universidad de San Jorge (No. 006–19/20).

## Results

Fifteen women completed the exercise protocol. The mean age of the patients was 53.5 ± 6.7 years. All participants had undergone surgery and hormonal treatment, and up to 66.6% had also received chemotherapy and radiotherapy. The mean time from diagnosis to the start of the program was 30.7 ± 19.4 months, with a range of 7–63 months.

### Subjective sleep assessment

At the end of our program, improvements were observed in several dimensions of sleep, as evaluated by the PSQI. The total PSQI score significantly decreased from 10.80 to 8.14, indicating an improvement in sleep quality (*p* = 0.003, effect size = 0.9). There were significant improvements in sleep latency (*p* = 0.035, effect size = 0.54), sleep duration (*p* = 0.011, effect size = 0.65), and sleep efficiency (*p* = 0.020, effect size = 0.60). No statistically significant changes were found in the use of sleep medications, subjective sleep quality, sleep disturbances, or daytime dysfunction. However, for the latter two dimensions, the effect sizes obtained were medium and large, respectively (see Table 1).

**Table 1:** PSQI scores before and after the exercise program

	Baseline Mean (SD)	Post Mean (SD)	Dif. baseline-post (95% CI)	*p*-value	Effect size
PSQI total	10.80 (4.60)	8.14 (4.66)	2.79 (1.13, 4.44)	**0.003**^**a**^	0.9^c^
Subjective sleep quality	1.47 (1.30)	1.14 (0.95)	0.36 (− 0.38, 1.09)	0.327^b^	0.25^d^
Sleep latency	2.13 (0.83)	1.64 (1.01)	0.50 (0.06, 0.94)	**0.035**^b^	0.54^d^
Sleep duration	2.13 (0.92)	1.64 (0.93)	0.57 (0.20, 0.94)	**0.011**^b^	0.65^d^
Habitual sleep efficiency	1.40 (1.35)	0.57 (0.76)	0.86 (0.18, 1.53)	**0.020**^b^	0.60^d^
Sleep disturbances	1.80 (0.56)	1.43 (0.51)	0.36 (− 0.07, 0.79)	0.096^b^	0.43^d^
Use of sleep medications	0.85 (1.16)	1.00 (1.24)	− 0.14 (− 0.78, 0.49)	0.680^b^	0.10^d^
Daytime dysfunction	1.07 (0.96)	0.79 (1.12)	0.21 (− 0.35, 0.78)	0.429^b^	3.06^d^

^a^Paired *t* test; ^b^Wilcoxon signed-rank test; ^c^Hedges’ g; ^d^*r* = *Z*/*n*

### Objective sleep assessment

The evaluation of sleep quality at the end of the program yielded two clinically relevant results. First, there was an increase in total sleep time (281.98 vs. 351.86 min, effect size = 1.00). Second, the average duration of awakenings slightly decreased (*p* = 0.021, effect size = 0.11) (see Table 2).

**Table 2:** Accelerometry results before and after the exercise program

	Baseline Mean (SD)	Post Mean (SD)	Dif. Baseline-Post (95% CI)	*p* value	Effect size
Sleep efficiency (%)	87.56 (3.17)	88.18 (3.95)	− 0.63 (− 3.25, 2.00)	0.649^a^	0.12^b^
Total sleep time (min)	281.98 (37.86)	351.86 (73.67)	− 69.89 (− 106.27, − 33.50)	0.088^a^	1.00^b^
Wake after sleep onset (min)	39.90 (10.87)	50.79 (20.92)	− 10.89 (− 22.03, 0.25)	0.227^a^	0.51^b^
Number awakenings	19.38 (4.90)	19.69 (6.89)	− 0.31 (− 3.79, 3.17)	0.073^a^	0.04^b^
Average awakenings length (min)	3.26 (0.68)	3.18 (0.86)	0.09 (− 0.31, 0.48)	**0.021**^**a**^	0.11^b^

^a^Paired *t* test; ^b^Hedges’ *g*

## Discussion

The results of the present pilot study showed that an online home-based exercise intervention can improve sleep quality and quantity in breast cancer survivors during lockdown.

In the context of the COVID-19 pandemic, patients with cancer have been particularly vulnerable to experiencing high levels of psychological distress and sleep disturbances. In fact, poor sleep quality has been identified as a common psychological symptom reported by patients with cancer during the COVID-19 pandemic [12]. Delays in diagnosis, changes in treatment, and access to healthcare services have contributed to sleep maintenance problems.

In the context of a lockdown, home-based exercise interventions can offer a practical and accessible approach to maintaining physical and psychological well-being for people affected by cancer. The results of this study also revealed the effectiveness of online home-based exercise interventions in improving sleep quality. Cancer patients undergoing active treatment are particularly susceptible to infections because of therapy-induced immunosuppression [13]. This heightened vulnerability necessitates preventive strategies that often constrain participation in conventional, in-person physical activity programs. Beyond the context of the COVID-19 pandemic, virtual exercise interventions have emerged as a feasible and safe alternative, offering immunocompromised individuals increased accessibility and reduced risk. Recent evidence supports the efficacy of group-based, videoconference-delivered exercise programs for cancer survivors, demonstrating high levels of feasibility, acceptability, and positive effects on physical activity engagement, fitness outcomes, and health-related quality of life [14]. These remote interventions confer multiple advantages, including the elimination of travel burdens, reduced exposure to clinical environments, and opportunities for tailored, supervised participation [15].

There are few studies examining the effects of home-based exercise programs on sleep quality in female breast cancer survivors. Türk et al. [16], in a study of breast cancer survivors (*n* = 45), implemented a 12-week unsupervised home-based aerobic program and reported positive changes in PSQI scores (not statistically significant). Similarly, Sprod et al. [17], in a study consisting of a home-based walking and resistance training exercise program during radiation therapy for breast and prostate cancer patients, reported a improvement in sleep quality in the exercise group (pre- to post-intervention), although the difference was not significant. This mixed evidence can be attributed to the diversity in types of exercise programs and the differing effects of exercise across different treatment protocols.

Ongoing cancer treatments have faced interruptions due to lockdowns, travel restrictions, or the reallocation of medical facilities for COVID-19 care, which has caused sleep dysfunction.

The use of objective measures such as accelerometry provides a robust and detailed understanding of how exercise can improve sleep quality in breast cancer patients. In our study, we used accelerometry to register many standard sleep parameters, as well as daily activity. We observed a positive effect of exercise on sleep efficiency and total sleep time. These results are in line with a review that highlighted how physical activity has a medium to large effect on improving sleep in cancer patients [18].

One of the key limitations of this study is that our pre-post intervention design is vulnerable to bias, therefore the impact of the intervention should be interpreted with caution. Other initiatives or information might have influenced our participants’ sleep, although to our knowledge, there were no other initiatives in this direction. In any case, our results are robust in describing sleep quality and quantity. Unlike previous studies, we evaluated sleep quality via a mixed subjective (PSQI) and objective (actigraphy) method. Both objective and subjective measurements are important for validating and determining the details of sleep quality. Thus, the combined results are more revealing and can also aid in personalized care to further improve the quality of life for cancer patients [19].

## Conclusion

Lockdowns can negatively impact the sleep quality of breast cancer survivors. Given the vulnerability of this population, strategies for promoting better sleep may include lifestyle modifications. A supervised online functional exercise program such as the present one may be a useful strategy to improve sleep quality in breast cancer survivors.

## Data Availability

The data supporting the findings of this study are provided within the article and its online supplementary materials. However, certain terms have been excluded to mitigate the risk of re-identification.
